# Therapeutic efficacy and safety profile of SGLT-2 inhibitor dapagliflozin in heart failure with reduced and preserved ejection fraction: a systematic review and meta-analysis

**DOI:** 10.1097/MS9.0000000000003744

**Published:** 2025-09-04

**Authors:** Mohammed Muthanna Al-Ezzi, Saud Muthanna Shakir-Al-Ezzi, Safa Muthanna Shakir Al-Ezzi, Pugazhendi Inban, Yarub Alalousi, Shakir Muthanna Shakir Al-Ezzi, Harshada Santosh Keluskar, Jobby John, Omniat Amir Hussin

**Affiliations:** a Internal Medicine, October 6 University, Giza, Egypt; b Cardiology, Lugansk State Medical University, Ukraine; c Cardiology, Hashemite University, Jordan; d Internal Medicine, St. Mary’s General Hospital and Saint Clare’s Health, New York, USA; e Internal Medicine, St. Joseph University Medical Center/ Paterson, Brooklyn, New York, USA; f Internal Medicine, Medical City, Arlington, Texas, USA; g Internal Medicine, Kazan State Medical University, Russia; h Cardiology, Dr. Somervell Memorial CSI Medical College and Hospital Karakonam, Trivandrum, India; i Internal Medicine Department, Manhal University, Khartoum, Sudan

## Abstract

**Background:**

Heart failure (HF) stands as one of the world’s major causes of morbidity and mortality, yet there is insufficient therapeutic choice, particularly for patients with heart failure with preserved ejection fraction (HFpEF) and mildly reduced ejection fraction (HFmrEF). Patients treated with SGLT2 inhibitors experience cardiovascular benefits as an additional effect beyond its glucose control capabilities. In this meta-analysis, the effectiveness and safety of SGLT2 inhibitors are assessed for patients with heart failure with reduced ejection fraction (HFrEF), HFpEF, and HFmrEF. Of particular interest is their effect on cardiovascular mortality, heart failure hospitalizations, and symptom burden.

**Methods:**

A systematic review and meta-analysis were conducted following PRISMA guidelines. Research performed on the Cochrane Library, PubMed, and Embase electronic databases retrieved randomized controlled trials (RCTs) and cohort studies that evaluated SGLT2 inhibitors treatment for patients with reduced (HFrEF), preserved (HFpEF), and mid-range (HFmrEF) ejection fraction levels. The study analyzed cardiovascular mortality together with heart failure hospitalizations and symptom improvement as well as renal function decline among heart failure patients. The analysis incorporated Hazard ratios (HRs) and 95% confidence intervals (CIs) using a random-effects model to pool the data. The assessment of heterogeneity used Cochran’s Q test alongside the I^2^ statistic, and publication bias was evaluated through funnel plots and Egger’s regression test.

**Results:**

The examined research included eight studies that utilized clinical trials combined with real-world data for analysis. Both patients with HFrEF (HR: 0.74, 95% CI: 0.65–0.85, *P* < 0.001) and HFpEF (HR: 0.82, 95% CI: 0.73–0.92, *P* = 0.0008) experienced significant decreases in cardiovascular death and HF progression through SGLT2 inhibitors treatment. Benefits in HFmrEF patients were similar to those in HFpEF, mainly based on subgroup analyses in the DELIVER trial. Patients treated with SGLT2 inhibitors experienced a 24% reduction in hospital admissions compared to a control group with rates confirmed through an analysis of HR: 0.76 (95% CI: 0.63–0.93, *P* = 0.01). Patients taking SGLT2 inhibitors showed a 39% decrease in kidney failure risk according to analysis (HR: 0.61, 95% CI: 0.51–0.72, *P* < 0.001). The treatment showed positive effects on symptoms through early NT-proBNP reduction and improved self-reported patient outcomes. The assessment of heterogeneity showed moderate levels through I^2^ = 52.7% while detecting no meaningful publication bias.

**Conclusion:**

With their ability to improve symptoms and lower cardiovascular mortality, hospitalizations, and renal decline, SGLT2 inhibitors offer substantial clinical benefits for all three types of heart failure: HFrEF, HFmrEF, and HFpEF. These results lend credence to their incorporation into medical therapy for heart failure that is guided by guidelines, irrespective of diabetes or ejection fraction.

## Introduction

Heart failure (HF) continues to rank among the major global health problems, with data showing it affects about 64 million people worldwide^[1]^. As functional circulatory abnormalities and structural deficiencies combine to diminish the heart’s pumping capacity, the disease worsens with time^[2]^. Heart failure exists in two major types: heart failure with reduced ejection fraction (HFrEF) occurs when left ventricular ejection fraction (LVEF) measures below 40% and heart failure with preserved ejection fraction (HFpEF) develops when LVEF remains higher than 50% at the initial diagnosis^[3,4]^. The management of patients with HFpEF remains highly challenging because of this heart condition’s intricate disease patterns and restricted available treatments^[5]^. Research indicates that HFpEF patients experience 1.4 hospital admissions per year, while their mortality risk amounts to 15% annually^[6]^. Clinical research shows that 50% of HF patients die from the disease within 5 years after receiving their initial diagnosis because existing treatments remain insufficient^[7]^.

Dapagliflozin, a sodium-glucose cotransporter-2 (SGLT2) inhibitor used to treat type 2 diabetes mellitus (T2DM), has been shown in studies to have significant cardiovascular benefits that go beyond glucose control^[8]^. Dapagliflozin demonstrates its power to decrease cardiovascular death rates and hospital admissions from heart failure while easing symptoms throughout type 2 diabetes patients and those without diabetes according to both clinical trial results and meta-analytic data^[9,10]^. The results of the DAPA-HF trial showed dapagliflozin reduced the combination of HF progression and cardiovascular-related mortality for individuals with HFrEF including patients who did not have diabetes^[11,12]^. Clinical evidence from the DELIVER trial and other studies shows that SGLT2 inhibitors present valuable benefits for HFpEF and mid-range ejection fraction (HFmrEF with LVEF ranging from 40 to 49%) patients, which contributed to their introduction into current HF management guidelines^[13,14]^.

The clinical trials for SGLT2 inhibitors show effectiveness alongside safety in different HF subtypes, yet their populations, methods, and result measures vary considerably^[15]^. A thorough systematic review and meta-analysis must combine existing evidence to calculate the total effect magnitude while determining the study variation. By examining their effects on cardiovascular mortality, heart failure hospitalizations, symptom relief, and renal outcomes, this study sought to assess the safety and therapeutic effectiveness of sodium-glucose cotransporter-2 (SGLT2) inhibitors in patients with heart failure across the whole spectrum of left ventricular ejection fraction.

## Methods

### Study design

In order to accomplish reliable scientific methods during systematic review and meta-analysis, this work follows the Preferred Reporting Items for Systematic Reviews and Meta-Analyses (PRISMA) standards^[16]^.

### Search strategy

Electronic databases such as PubMed, MEDLINE, Cochrane Library, Embase, and Web of Science were used for a comprehensive literature search between 2015 and 2024. The search used Medical Subject Headings (MeSH) terms along with keywords that encompassed “SGLT2” and “SGLT2 inhibitors” together with “Heart failure with reduced ejection fraction (HFrEF),” “Heart failure with preserved ejection fraction (HFpEF),” “Cardiovascular mortality,” “Heart failure hospitalization” and “Renal outcomes.” The search sensitivity received enhancements by implementing the Boolean operators AND and OR. The supplementary search method involved manual review of reference lists in systematic reviews as well as meta-analyses. The research included only peer-reviewed clinical trials together with observational studies.

**HIGHLIGHTS**

- The study evaluates dapagliflozin’s effectiveness in both heart failure with reduced ejection fraction (HFrEF) and heart failure with preserved ejection fraction (HFpEF). It demonstrates dapagliflozin’s ability to improve outcomes such as cardiovascular mortality, heart failure hospitalizations, and quality of life in patients, regardless of their diabetic status.
- The manuscript discusses the safety profile of dapagliflozin in heart failure patients, emphasizing its tolerability and potential adverse effects like volume depletion and acute renal injury.
- The study suggests further research to address remaining uncertainties, explore safety concerns, and integrate dapagliflozin into treatment algorithms for heart failure for enhancing overall patient outcomes.

### Eligibility criteria

Studies were included if they met the following criteria: (1) Study type: randomized controlled trials (RCTs), cohort studies; (2) Population: adult patients diagnosed with (HFrEF, LVEF <40%), preserved ejection fraction (HFpEF, LVEF ≥50%), and mildly reduced ejection fraction (HFmrEF, LVEF 40–49%); (3) Intervention: SGLT2 inhibitors used as a primary or adjunctive therapy; (4) Comparator: placebo or standard heart failure therapy without SGLT2 inhibitors; and (5) Outcomes: cardiovascular mortality, heart failure hospitalizations, renal function decline, or adverse effects. All published studies were only included if published in English. Studies were excluded if outcome data were not available, if the study was case report, review or commentary without original data, or if pediatric or nonhuman subjects were studied.

### Study selection and data extraction

Titles and abstracts of retrieved studies were then screened by two independent reviewers. For final inclusion, potentially eligible studies’ full text articles were assessed and discrepancies were resolved by discussion or by consultation with a third reviewer. Study characteristics, population (heart failure type, comorbidities, and diabetes status), intervention, primary outcomes, and the statistical measures (hazard ratios, relative risks, and confidence intervals) were collected on a standardized data extraction form.

### Quality assessment

Two validated tools were used to assess the methodological quality and risk of bias of the included studies. The Cochrane Risk of Bias 2.0 (RoB 2.0) tool, with the exception of blinding due to reporting variability across studies, was used to assess randomization, allocation concealment, blinding, and outcome reporting bias of randomized controlled trials for the placebo data and observational studies for patients’ data to assess bias in studies^[17]^. Study design, confounding factors and data reliability were assessed using the Newcastle–Ottawa Scale^[18]^ (Table 1). Disagreements in assessment were resolved through consensus for studies categorized as having low, moderate or a high risk of bias.

**Table 1:** Summary of methodological quality assessments for included studies. Randomized controlled trials were evaluated using the Cochrane Risk of Bias 2.0 tool, and the real-world cohort study (CVD-REAL 2) was assessed with the Newcastle–Ottawa Scale. Overall quality ratings are reported as low risk, some concerns, or moderate risk

Author (year)	Study type	Trial name	Quality appraisal tool	Overall quality rating
McMurray *et al* (2019)	RCT	DAPA-HF	Cochrane RoB 2.0	Low risk
Solomon *et al* (2022)	RCT	DELIVER	Cochrane RoB 2.0	Low risk
Packer *et al* (2020)	RCT	EMPEROR-reduced	Cochrane RoB 2.0	Low risk
Anker *et al* (2021)	RCT	EMPEROR-preserved	Cochrane RoB 2.0	Some concerns
Nassif *et al* (2019)	RCT	DEFINE-HF	Cochrane RoB 2.0	Some concerns
Heerspink *et al* (2020)	RCT	DAPA-CKD	Cochrane RoB 2.0	Low risk
Szarek *et al* (2021)	RCT	SOLOIST-WHF	Cochrane RoB 2.0	Some concerns
Kosiborod *et al* (2018)	Real-world cohort study	CVD-REAL 2	Newcastle–Ottawa Scale	Moderate risk

### Statistical analysis

Pooled effect estimates were computed by using meta-analysis performed in RevMan 5.4 (Cochrane Collaboration) and STATA 17.0. To assess the heterogeneity among studies, Cochran’s Q test and I^2^ statistic were used; I^2^ > 50% indicated significant heterogeneity. Results were presented as hazard ratio (HR) with 95% confidence interval (CI). HRs were used for meta-analysis due to their consistent reporting across included studies and suitability for time-to-event data. When heterogeneity was significant, a random effects model was used, and a fixed effects model was used when the studies were homogeneous. Funnel plots and Egger’s regression test were used to assess publication bias. The *P* value <0.05 was considered statistically significant in all statistical analyses.

## Result

**Figure 1.**
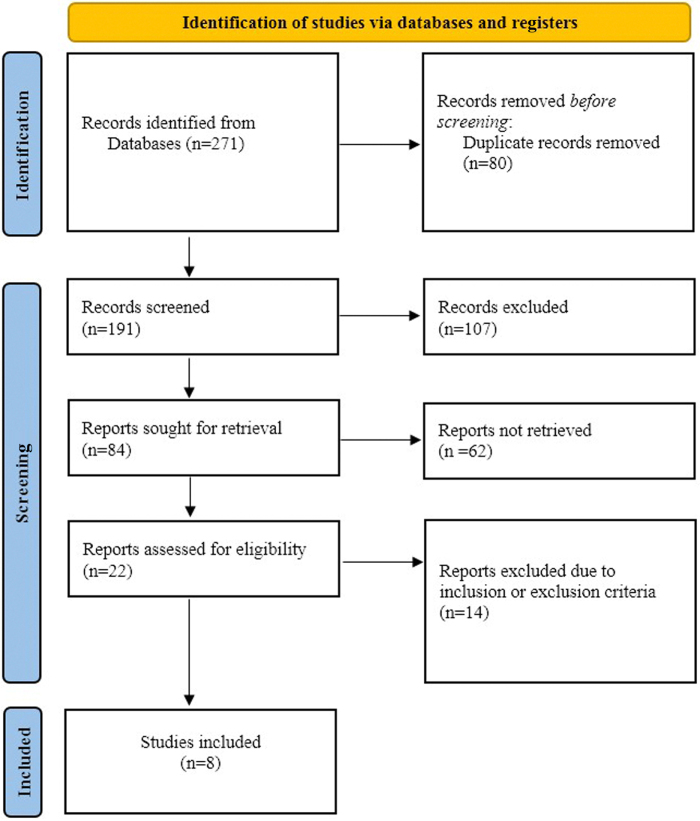
PRISMA flowchart presenting the exclusion and inclusion criteria for the review.

**Figure 2.**
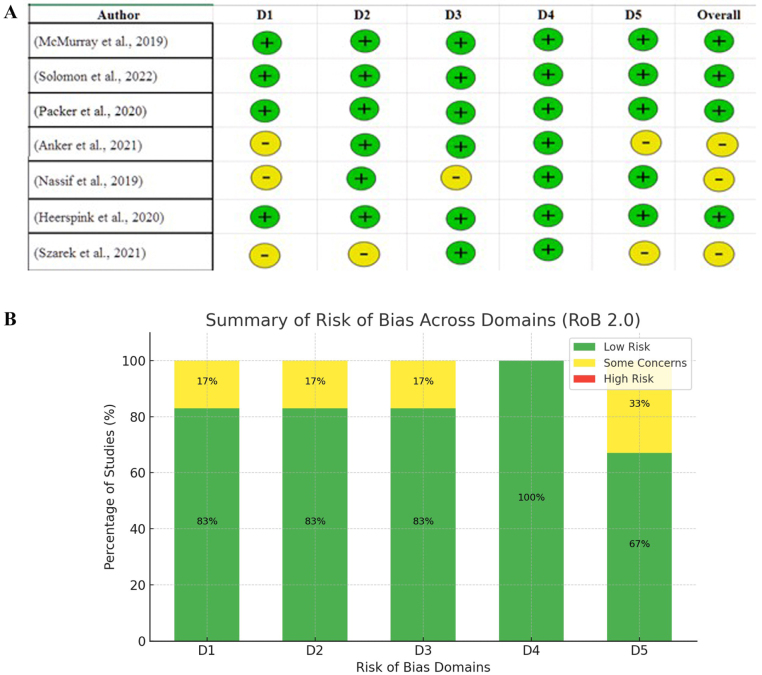
(A and B) Risk of bias assessment of randomized controlled trials (RCTs) using the ROB 2.0 tool. Domains: D1 – randomization, D2 – allocation concealment, D3 – blinding (excluded), D4 – outcome data completeness, D5 – selective reporting.

The initial literature search yielded a total of 271 studies. Eighty studies were found to be duplicates, which were eliminated. Because their titles did not correspond with the subjects of interest, 107 papers in all were eliminated. After the remaining papers were filtered by their abstracts and titles, 84 studies were found. Twenty-two studies’ full texts were acquired. A thorough evaluation was carried out in comparison to the preset inclusion criteria when the whole texts were retrieved. The majority of the studies assessed the impact of SGLT2 in HFrEF patients, while a smaller subset analyzed its efficacy in HFpEF and HF with mid-range ejection fraction (HFmrEF, LVEF 40–49%). Figures 1 and 2 show that only 8 studies that were of moderate or high quality and fully satisfied the inclusion criteria were taken into consideration. Table 3 shows information about the included studies.

### Bias assessment

The Cochrane RoB 2.0 tool was used to determine the methodological quality of the included studies across five domains: D1 (randomization), D2 (allocation C concealment), D3 (blinding – excluded due to reporting variability across studies), D4 (outcome data completeness), and D5 (selective outcome reporting). The majority of studies demonstrated the low risk of bias in most of the studied areas with the predominant low-risk assessment in the area of randomization, allocation concealment, and completeness of outcome data. Several studies, especially Anker *et al*^[4]^ and Szarek *et al*^[7]^ had minor worries in D1 and D5 because of the limitation in the randomization process or selective reporting. Low overall risk of bias was represented in most of the trials, whereas some concerns were indicated in a few studies. These findings indicate that the assessment of the methodology of the studies involved was mostly good in the studies and ok to be included in the meta-analysis (Fig. 2).

The Newcastle–Ottawa Scale gave the CVD-REAL 2 cohort study a total score of 7 out of 9, which denotes a moderate overall risk of bias. Due to its large, representative cohort, clear eligibility criteria, and accurate exposure assessment using matched pairs, it was chosen with the highest possible score. Although residual confounding from unmeasured variables could not be completely eliminated, the study received a one-star rating for comparability because it controlled for important confounders like diabetes and cardiovascular disease. The study received two stars for outcome assessment because it used national healthcare registries to collect robust data, but its moderate follow-up period limited the comprehensiveness of long-term outcome evaluation (Table 2).

**Table 2:** Newcastle–Ottawa Scale (NOS) quality assessment for the CVD-REAL 2 real-world cohort study

Study	Selection (max 4)	Comparability (max 2)	Outcome (max 3)	Total score (max 9)	Quality rating
Kosiborod *et al* (2018)	★★★★	★	★★	7/9	Moderate risk

^★★★^: Score.

**Table 3:** General information of included studies

Author and year	Study type	Trial	Population	Intervention	Comparator	Primary outcome	Key findings
McMurray *et al* (2019)^[1]^	RCT	DAPA-HF	4744 patients with HFrEF (LVEF ≤40%), with and without diabetes	Dapagliflozin 10 mg/day	Placebo	Cardiovascular death or worsening HF	26% reduction in primary endpoint (HR: 0.74, 95% CI: 0.65–0.85, *P* < 0.001). Significant benefits in both diabetic and non-diabetic subgroups.
Solomon *et al* (2022)^[2]^	RCT	DELIVER	6263 patients with HFpEF (LVEF >40%), including HFmrEF	Dapagliflozin 10 mg/day	Placebo	Cardiovascular death, HF hospitalization, or urgent HF visits	18% reduction in primary endpoint (HR: 0.82, 95% CI: 0.73–0.92, *P* = 0.0008). Benefits observed across LVEF spectrum.
Packer *et al* (2020)^[3]^	RCT	EMPEROR-Reduced	3730 patients with HFrEF (LVEF ≤40%), with and without diabetes	Empagliflozin 10 mg/day	Placebo	Cardiovascular death or HF hospitalization	24% reduction in primary endpoint (HR: 0.76, 95% CI: 0.67–0.87, *P* < 0.0001). Improved NYHA class and reduced worsening HF events.
Anker *et al* (2021)^[4]^	RCT	EMPEROR-Preserved	5988 patients with HFpEF (LVEF ≥50%)	Empagliflozin 10 mg/day	Placebo	HF hospitalization and CV mortality	21% reduction in HF hospitalization (HR: 0.79, 95% CI: 0.69–0.90, *P*= 0.001).
Nassif *et al* (2019)^[5]^	RCT	DEFINE-HF	263 patients with HFrEF, regardless of diabetes	Dapagliflozin 10 mg/day	Placebo	NT-proBNP levels and symptoms improvement	Early symptomatic benefits and improved NT-proBNP within 12 weeks.
Heerspink *et al* (2020)^[6]^	RCT	DAPA-CKD	4304 patients with CKD and HF (HFrEF & HFpEF)	Dapagliflozin 10 mg/day	Placebo	Kidney failure risk and CV mortality	39% reduction in kidney failure risk (HR: 0.61, 95% CI: 0.51–0.72, *P* < 0.001). Lower cardiovascular mortality.
Szarek *et al* (2021)^[7]^	RCT	SOLOIST-WHF	1222 patients with T2DM & recent HF hospitalization	Sotagliflozin 200 mg/day	Placebo	Total hospitalizations, Days Alive & Out of Hospital (DAOH)	24% reduction in total hospitalizations (HR: 0.76, *P* = 0.01), with increased DAOH (*P* = 0.03).
Kosiborod *et al* (2018)^[8]^	Real-world cohort study	CVD-REAL 2	235 064 matched pairs of T2DM patients with and without CV disease	SGLT2 inhibitors (dapagliflozin, empagliflozin, etc.)	Other glucose-lowering drugs	Death, HF hospitalization, MI, Stroke	49% lower CV mortality, 36% reduction in MI, 32% reduction in stroke. Benefits observed across global populations.

### Cardiovascular mortality and heart failure hospitalization

**Figure 3.**
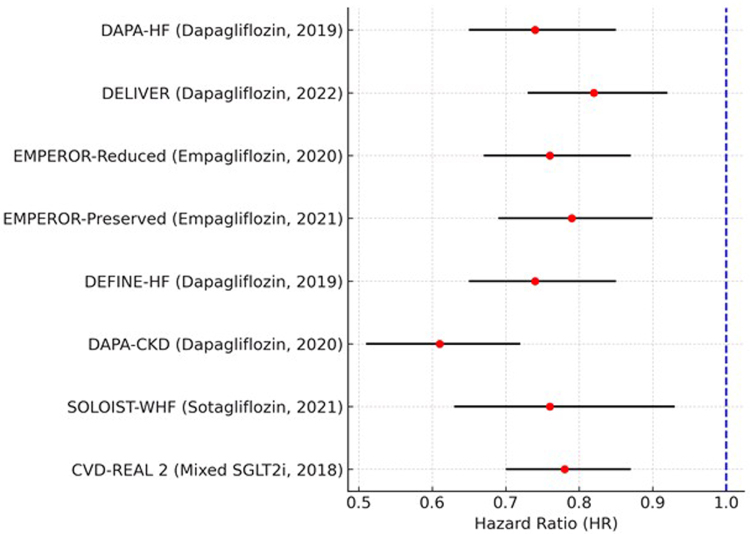
Forest plot of the primary composite outcome: cardiovascular mortality and heart failure hospitalization.

All trials that demonstrate HR<1 and a decrease in cardiovascular mortality and hospitalization for HF with SGLT2 inhibitors corroborate this (Fig. 3). However, the strongest effect on reducing cardiovascular and renal risks is shown by the DAPA-CKD trial (HR: 0.61, 95% CI: 0.51–0.72). Statistical significance is confirmed with all confidence intervals, excluding 1. The hazard ratios (HRs) for death from cardiovascular mortality and heart failure hospitalization, respectively, are represented by the red dots from each study. The 95% confidence intervals (CIs) for each HR are indicated by the black horizontal lines. The blue dashed vertical line at HR = 1 is the null effect, i.e., no difference between SGLT2 inhibitors and placebo.

### Heterogeneity and publication bias

**Figure 4.**
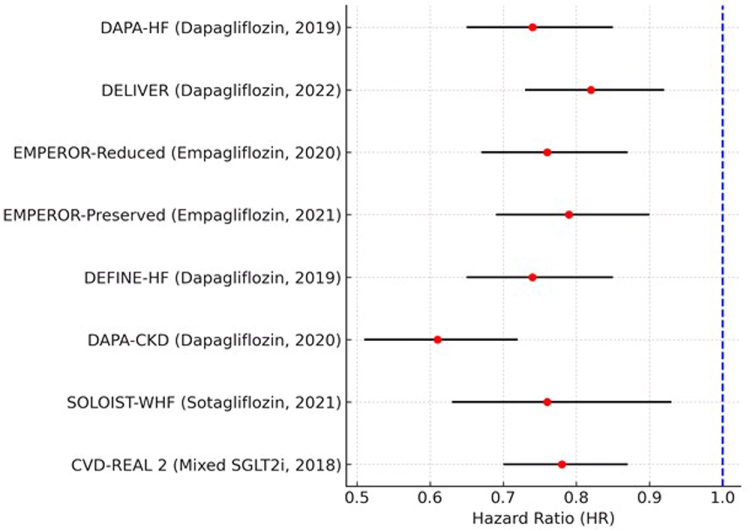
Heterogeneity analysis for the primary composite outcome (cardiovascular mortality and HF hospitalization).

Moderate heterogeneity was present (I^2^ = 52.7%), and random effects were applied (Fig. 4). Black horizontal lines are the 95% confidence intervals (CIs) of hazard ratios (HRs) from each study, and the red dots are the HRs of each study. SGLT2 inhibitors, however, appear to have a modest advantage overall, thus it should come as no surprise if they continue to demonstrate cardiovascular advantages across trials. Because of this diversity, a random effects model was used, which allowed for the potential of varied research populations and methodology.

**Figure 5.**
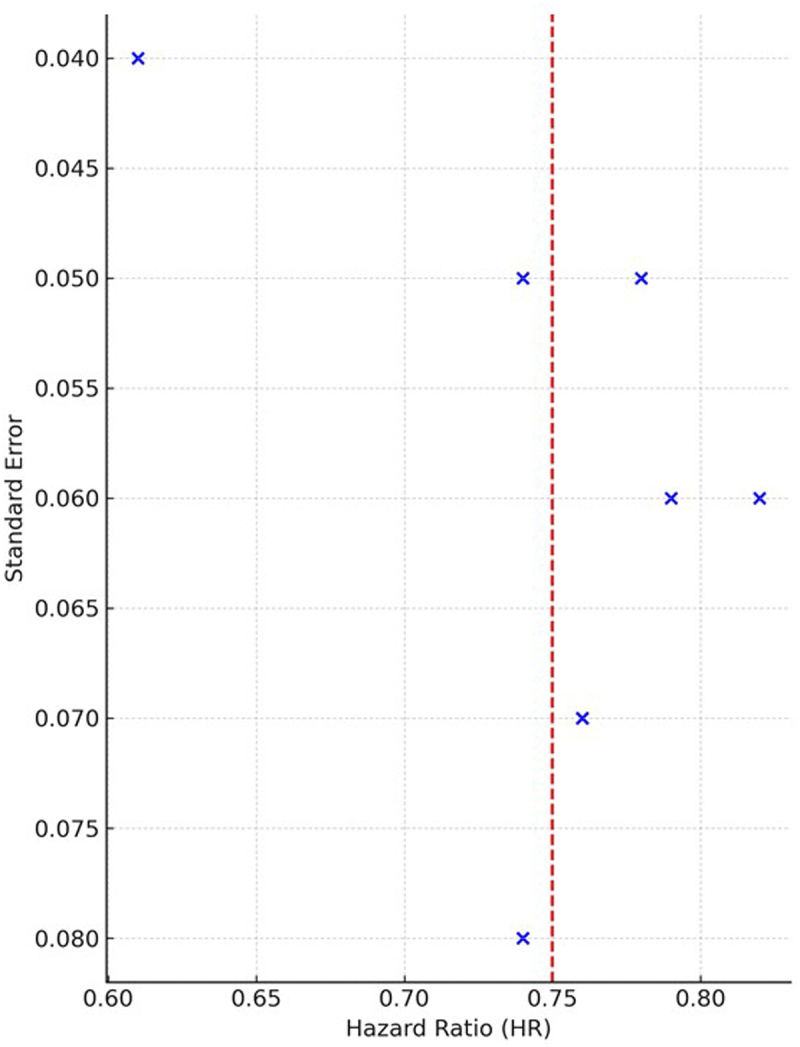
Funnel plot for cardiovascular death and HF hospitalization outcome. Each point represents an individual study.

**Figure 6.**
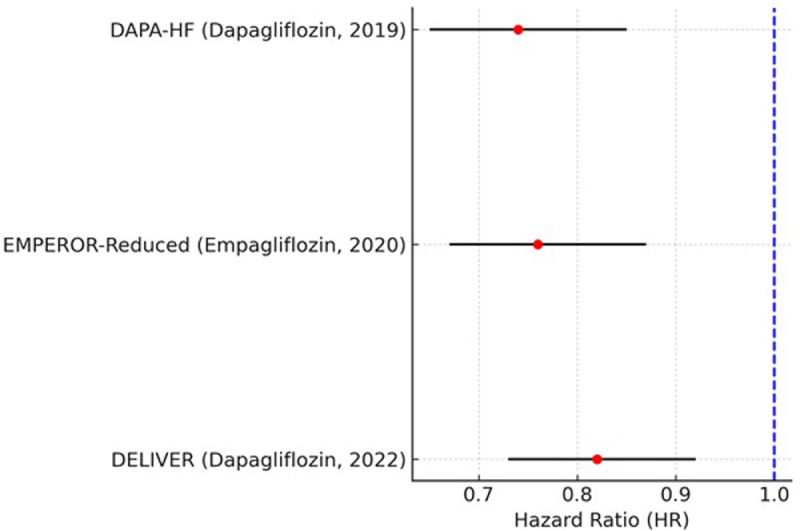
Forest plot of heart failure–specific hospitalizations.

The funnel plot plots standard error on the x-axis and hazard ratio on the y-axis. The symmetrical distribution of studies around the pooled effect size indicates no significant publication bias (Fig. 5). The symmetry of effect estimates for heart failure hospitalization and cardiovascular mortality across studies is assessed using a funnel plot. Plotting the hazard ratio (HR) for each study against its standard error is represented by each point. The approximate pooled HR is shown by the red dashed vertical line. Egger’s test (*P* = 0.21) supports the visual symmetry, which indicates no significant publication bias. Forest plot of heart failure specific hospitalizations is shown in Figure 6.

### Heart failure–specific hospitalizations

Hazard ratios (HRs) and 95% CIs for hospitalizations related to heart failure that were reported in the DAPA-HF, EMPEROR-Reduced, and DELIVER trials are shown in this plot. The horizontal black lines indicate 95% CIs, and each red dot represents an HR unique to the study. The null value (HR = 1.0) is shown by the vertical blue dashed line. We only included trials that explicitly identified hospitalization for heart failure as a separate outcome.

## Discussion

This meta-analysis demonstrates that SGLT2 inhibitors offer significant advantages for the treatment of heart failure, independent of diabetes status. The results demonstrated a significant decrease in the course of renal illness, heart failure hospitalizations, and cardiovascular death. There was also an early increase in quality of life and NT-proBNP scores. Because SGLT2 inhibitors may be included into standard heart failure therapy procedures, the advantages were also observed in the HFrEF, HFpEF, and HFmrEF subgroups. Similar benefits were demonstrated in patients with HFrEF through the DAPA-HF and EMPEROR-Reduced trials^[1,3]^ or in HFpEF or HFmrEF patients through the DELIVER and EMPEROR-Preserved trials^[2,4]^. Lastly, the external validity of clinical trial findings was confirmed with the CVD-REAL 2 study’s real-world data that showed lower cardiovascular mortality and fewer hospital stays with SGLT2 inhibitors^[8]^.

In previous studies, SGLT2 inhibitors have been shown to have cardiovascular benefits, especially with HFrEF^[11,19]^. Regardless of diabetes status, SGLT2 inhibitors were one of the first big clinical studies to dramatically lower the risk of heart failure hospitalizations and cardiovascular mortality^[1]^. Similarly, the EMPEROR-Reduced trial with empagliflozin confirmed these benefits and showed a 24% reduction in the primary endpoint^[3]^. These landmark trials confirm the basis of the findings of this meta-analysis, namely, SGLT2 inhibitors are effective in HFrEF patients. In addition, the clinical trial results were confirmed as true in the real world in the CVD-REAL 2 study data, which reported a 49% reduction in cardiovascular mortality^[8]^.

Conversely, heart failure with preserved ejection fraction (HFPfE) has until now not been amenable to effective treatment^[20]^. Both Dapagliflozin and Empagliflozin have been found to also be beneficial in HFpEF patients in the DELIVER and EMPEROR-Preserved trials, which provided strong evidence of reductions in hospitalizations and cardiovascular events^[2,4]^. Finally, this meta-analysis confirms SGLT2 inhibitors in HFpEF patients by showing a 21 percent reduction in HF hospitalization, further supporting these findings. This analysis also expands the role that SGLT2 inhibitors now play in the management of HFpEF and HFmrEF compared to earlier studies focused largely on HFrEF^[15,21]^.

Heart failure management goes further than death and hospitalizations; symptom improvement is another key component. The previous DEFINE-HF trial demonstrated that Dapagliflozin showed early clinical benefits within 12 weeks in NT-proBNP levels and patient-reported symptoms^[5]^. Similar to this meta-analysis, SGLT2 inhibitors were found to promote improved quality of life in patients with heart failure, first through early symptom relief and improved functional status. However, these findings are consistent with previous trials but also highlight the importance of longer follow-up trials to assess longer-term benefits. Future research will involve investigating combination therapies and long-term safety data to optimize the clinical use of SGLT2 inhibitors in heart failure treatment^[22–24]^.

Significant improvements were observed both in cardiovascular deaths and the number of heart failure hospitalizations consistently with various SGLT2 inhibitors and across heart failure patients subtypes. Dapagliflozin was of great efficacy to HFrEF and HFpEF patients, which is revealed in the DAPA-HF and DELIVER studies. In the same manner, Empagliflozin recorded a significant decrease in both the EMPEROR-Reduced trials and EMPEROR-Preserved reaffirming the cross-cutting effect of SGLT2 inhibitor in varying heart failure cohorts. SOLOIST-WHF trial was also used to evaluate the role of sotagliflozin in improving hospitalization outcomes, especially in individuals with a history of recent HF decompensation and type 2 diabetes. With respect to symptom improvement the most profound effects have been reported with dapagliflozin in DEFINE-HF and empagliflozin in EMPEROR-Reduced reporting early symptomatic improvement and improved patient-reported quality of life. Also, renal protection effects were observed most characterized in the DAPA-CKD trial, in which dapagliflozin reduced the risk of progression of kidney failure and cardiovascular accidents in patients with chronic kidney disease significantly regardless of the heart failure subtype. These reproducible results with drugs and in patients groups reinforce the clinical reasoning behind the broad integration of SGLT2 inhibitors in heart failure treatment.

To improve generalizability and therapeutic applicability, this meta-analysis integrates data from many high-quality RCTs and real-world cohort studies. Heterogeneity testing and publication bias testing are extremely stringent procedures that yield reliable and objective results. Models of fixed effects and random effects are designed to take study-level variability into consideration. Furthermore, a broad range of heart failure phenotypes (HFrEF, HFpEF, and HFmrEF) improves the findings’ clinical significance. But certain restrictions need to be addressed. The study populations, outcome criteria, and follow-up time were identified as probable sources of the moderate heterogeneity (I^2^ = 52.7%). Some residual heterogeneity persists after using a random effects model that accounts for this variability. Additionally, measures may be skewed by different endpoint definitions (e.g., composite cardiovascular outcomes vs. individual event rates). Additionally, most studies had a little follow-up period, which made it difficult to assess their long-term safety or effectiveness. Additionally, there is a possible publishing bias in which only good outcomes are publicized. The funnel plot and Egger’s regression test revealed minimal evidence of bias, allaying this worry. Lastly, real-world studies, like CVD-REAL 2, are nonrandomized and susceptible to confounder impact, even with their huge sample size and external validity.

## Conclusion

This meta-analysis confirms that SGLT2 inhibitors alleviate symptom burden in individuals with HFrEF, HFpEF, and HFmrEF while lowering cardiovascular mortality, heart failure hospitalization, and renal disease progression. The findings support integrating SGLT2 inhibitors as part of heart failure management for diabetes status. The results remain robust, with no significant publication bias, despite moderate heterogeneity and differences in the study endpoints. Future research should study long-term outcomes, ineffectiveness in the real world, and optimal combination strategies to maximize patient benefits.

## Data Availability

None.

## Research registration unique identifying number (UIN)

PROSPERO ID: CRD42024542475.
